# Effect of metabolic control on oxidative stress, subclinical atherosclerosis and peripheral artery disease in diabetic patients

**DOI:** 10.1186/s40200-015-0215-5

**Published:** 2015-11-10

**Authors:** Ozra Tabatabaei-Malazy, Hossein Fakhrzadeh, Farshad Sharifi, Mojde Mirarefin, Seyed Masoud Arzaghi, Zohre Badamchizadeh, Mahtab Alizadeh Khoee, Bagher Larijani

**Affiliations:** Diabetes Research Center, Endocrinology and Metabolism Clinical Sciences Institute, Tehran University of Medical Sciences, Tehran, Iran; Endocrinology and Metabolism Research Center, Endocrinology and Metabolism Clinical Sciences Institute, Tehran University of Medical Sciences, Tehran, Iran; Elderly Health Research Center, Endocrinology and Metabolism Population Sciences Institute, Tehran University of Medical Sciences, Tehran, Iran

**Keywords:** Diabetes, Oxidative stress, Subclinical atherosclerosis, Peripheral artery disease

## Abstract

**Introduction:**

By rising diabetes mellitus prevalence, the prevalence of its most complication; cardiovascular disease (CVD) is also increasing. Moreover, oxidative stress has important role in pathogenesis of diabetes and its complications. We investigated relationship between total antioxidant status (TAS) and surrogate measures of subclinical atherosclerosis (SA) with glycemic status in diabetics.

**Methods & materials:**

In a cross-sectional study, we recorded height, weight, waist circumference (WC) and blood pressure of 267 subjects. Blood samples were collected to measure fasting blood sugar (FBS), glycated hemoglobin (HbA1c), lipid profiles and TAS. The surrogate measures of SA were Carotid Intima Media Thickness (CIMT), and Ankle Brachial Index (ABI).

**Results:**

We found significantly lower TAS leves and ABI values and higher CIMT in diabetic patients especially in poor glycemic group. There was a nonsignificant, weak correlation between TAS, ABI and CIMT with glycemic status (*r* = −0.10, −0.16, and +0.09, respectively). Multivariate regression analysis showed a significant influence of increasing age and diabetes duration on worsening CIMT in poor glycemic group.

**Conclusions:**

Our study showed poor glycemic control leads to worse CIMT by increasing age and duration of diabetes. However we did not find a significan correlation between glycemic status and TAS levels. We suggest CIMT measurement along with other SA markers in poor glycemic diabetics, especially in older patients with longer duration of diabetes, to identify high risk CVD patients.

## Background

Diabetes mellitus is a chronic hyperglycemic state associated with serious cardiometabolic abnormalities such as insulin resistance, hyperlipidemia, hypertension, and obesity. These aberrancies are almost always accompanied by oxidative stress [1]. A significant reduction in the efficiency of antioxidant defenses and/or overproduction of reactive oxygen species (ROS) [2] has a crucial role in the development of the diabetes complications [2]. In diabetic patients, oxidative stress is evident within a few years after involvement and before complications become manifest [3].

Cardiovascular disease (CVD) is the most threatening complication of diabetes. While the leading cause of mortality worldwide, it is three to four times more common in diabetics than non-diabetics individuals [4, 5]. On the other hand it is established that the diabetes itself is an independent risk factor for accelerating of atherosclerosis. The role of hyperglycemia as an independent risk factor for development of CVD is supported by the United Kingdom Prospective Diabetes Study (UKPDS) [6]. As the prevalence of DM is rising, it will be a major contributory risk factor of increased CVD events. Like other developing countries we are confronting an escalating trend of CVD in Iran [7]. So measurement of surrogate markers of atherosclerosis is of utmost importance for circumventing CVD end-points, especially in diabetics subjects. A reliable measure of metabolic control in diabetics is glycosylated hemoglobin (HbA1c) which reflects recent mean glucose concentration over the past 3 months and provides a relatively substantial base for prediction of CVD events [8, 9]. It is expected that improving glycemic control with HbA1c would delay the onset and ameliorate the severity of diabetic complications [6].

It is possible to evaluate plasma markers of oxidative stress. One of oxidative stress indicators is total antioxidant status (TAS) which represents the plasma level of cumulative antioxidant reserve of the body and enables the evaluation of the average antioxidant potential [10]. Conflicting reports on serum concentration of TAS in patients with CVD have been reported in epidemiological studies [11]. However, given its feasibility, measurement of TAS can be used as a valuable tool for investigating the association between dietary antioxidant status and CVD [12].

Atherosclerosis is the most common type of CVD. There are several non invasive methods to assess subclinical atherosclerosis (SA) including Carotid Intima Media Thickness (CIMT) [13], and Ankle Brachial Index (ABI) [14]. CIMT is a valid surrogate marker of CVD that can detect intimal atherosclerotic process and medial hypertrophy in carotid arteries. Increased CIMT is an early phenomenon in development of atherosclerosis even in asymptomatic individuals [13].

It is established both asymptomatic and symptomatic PAD can increase the risk of CVD. Ankle brachial index (ABI) is a simple, inexpensive, and noninvasive measure and also sensitive and cost-effective screening tool for peripheral artery disease (PAD) [15]. ABI can also provide useful information about cardiovascular health both in general population and in clinical settings [15].

There are various reports about the effect of glycemic control on CVD risk reduction in type 2 diabetes mellitus (T2DM) [16, 17]. However, we didn’t find any study to assess the association between glycemic status with oxidative stress and surrogate measures of CVD in T2DM. This cross-sectional study was designed to determine the effect of metabolic control on plasma status of TAS and surrogate measures of atherosclerosis in T2DM.

## Methods

### Study population

We conducted a cross-sectional study in 267 participants; 150 non-diabetics and 117 T2DM patients during a period of 12 months between January 1^th^ and December 31^th^2012 in diabetes clinic of Dr. Shariati Hospital, Tehran/ Iran. T2DM was diagnosed according to American Diabetes Association criteria [9]. Inclusion criteria were T2DM or non-diabetics aged between 35–75 years (mean ± SD 51.5 ± 8.1 years). Subjects who ingested antioxidant supplements and drugs in last 3 months ago, suffering from acute or chronic inflammatory/ infectious conditions, past medical record of CVD or acute myocardial infarction, and chronic diseases such as chronic renal failure, or cirrhosis were excluded from the study. After obtaining the written consent of participants, T2DM patients were divided into two groups of good control (HbA1c ≤ 7.5 %) and poor control (HbA1c > 7.5 %) according to their glycemic status.

A personal and demographic questionnaire was filled for all of the patients. Height, weight, waist circumference (WC) and blood pressure were measured with standard tools and recorded. Body mass index (BMI) was calculated by deviding weight (kg) to height squared (m)^2^. WC was measured on broadest area between the edge of lower ribs and the iliac crest in standing position. Systolic and diastolic blood pressures were measured after 5–10 min rest, on the right arm in sitting position. Hypertension was defined as systolic blood pressure/ diastolic blood pressure ≥ 140/90 mmHg or under treatment with antihypertensive medications.

### Laboratory measures

Blood samples were collected in fasting state and were analyzed for fasting blood sugar (FBS), total cholesterol (TC), triglycerides (TG) and high density lipoprotein cholesterol (HDL-C) by autoanalyser, Pars Azmoon Kit (Iran). The low density lipoprotein cholesterol (LDL-C) was derived by Fredrickson-Friedwald’s formula [LDL-C = (TC– HDL-C)– TG/5], if TG level was < 400 mg/dl [18]. Dyslipidemia was defined as TC ≥ 200, TG ≥ 150 mg/dl, LDL-C > 100 mg/dl, HDL-C < 40 mg/dl in men and < 50 mg/dl in women based on definition of dyslipidemia in NCEP ATP III^1^ [19] or taking anti-hyperlipidemic medications. HbA1c was measured by DS5 chromatography; Drew Scientific Limited Company (UK). TAS levels were measured by ELISA kits [Cayman (US)].

### Subclinical atherosclerosis measures

We measured CIMT and ABI for detection of subclinical atherosclerosis. Ultrasonographic analysis of the carotid arteries was performed with a high-resolution ultrasound scanner, equipped with a linear array 13 MHz transducer (MyLab 70 X vision, biosound esaote USA). For detection of CIMT special software (vascular tools 5, Medical Imaging Applications LLC, USA) was employed. The means of the three maximum right and three maximum left far wall measurements of the proximal part, mid part and the bulb were calculated for each common carotid artery. In our study, all the six right and left wall values were measured and the average values noted. The cut off point for SA definition by CIMT was considered an extreme increase of common carotid IMT ≥ 0.8 mm [20]. ABI was measured as the ratio of the average systolic blood pressure at the ankle of each leg divided by the average systolic blood pressure in the arm. Then the highest blood pressure of limbs considered. The ABI ≤ 0.9 was considered diagnostic for PAD [15].

This study was approved by the Ethics Committee of Tehran University of Medical Sciences of Tehran/ Iran.

### Statistical analysis

The normal distribution of data was verified by Kolmogrov-Smirnov analytic test. All variables had normal distribution except TG, TAS, and CIMT. Paired T-Test was applied to variables with normal distribution, and Wilcoxon and Mann–Whitney nonparametric tests for analysis of following parameters: TG, TAS, and CIMT. Also, we used univariate and multivariate logistic regression models according to HbA1c categorized status (<7.5/ ≥7.5 %) adjusted for sex, age, duration of diabetes, BMI, WC, hypertension, and hyperlipidemia. Outcome variables included plasma levels of TAS, and surrogate measures of SA, that were CIMT and ABI. All independent variables which had *p* < 0.2 in the univariate model were selected for the multivariate analysis. The p value ≤ 0.05 was considered as statistically significant.

## Results

The baseline characteristics of all participants are shown in Table 1.

**Table 1:** Baseline characteristics of participants in control and diabetes groups

Variable	Control group *N* = 150	Diabetes group *N* = 117	*P* value
Age (year)	49.8 ± 7.5	53.8 ± 8.3	<0.001*
Female (n (%))	87 (58)	61 (52.1)	0.38
Diabetes duration (year)	------	8.9 ± 6.6	-----
SBP (mmHg)	126 ± 17	134 ± 18	<0.001*
DBP (mmHg)	78 ± 11	78 ± 10	0.93
BMI (kg/m^2^)	28.4 ± 4.3	28 ± 4.3	0.41
WC (cm)	92 ± 10.7	94.8 ± 10.8	0.04*
TC (mg/dl)	200 ± 34	176 ± 42	<0.001*
TG (mg/dl)^a^	161 ± 80	193 ± 109	0.002*
LDL-C (mg/dl)	114 ± 23	95 ± 26	<0.001*
HDL-C (mg/dl)	46 ± 10	42 ± 9	<0.001*
FBS (mg/dl)	95 ± 12	166 ± 63	<0.001*
HbA1c (%)	5.3 ± 0.6	8 ± 1.8	<0.001*
TAS (mmol/l)^a^	3.2 ± 0.8	3.1 ± 0.6	0.02*
Max. CIMT (mm)^a^	1 ± 0.5	1.3 ± 1	<0.001*
ABI	1.17 ± 0.11	1.13 ± 0.11	0.003*

*SBP* systolic blood pressure, *DBP* diastolic blood pressure, *BMI* body mass index, *WC* waist circumference, *TC* total cholesterol, *TG* triglycerides, *LDL-C* low density lipoprotein cholesterol, *HDL-C* high density lipoprotein cholesterol, *FBS* fasting blood sugar, *HbA1c* glycated hemoglobin A1c, *TAS* total antioxidant status, *Max. CIMT* maximum carotid intima media thickness, *ABI* ankle brachial index

*P ≤ 0.05 was considered as statistically significant

^a^For variables with abnormal distribution nonparametric analytic tests were used

Of our diabetic patients, 7 patients (6 %) were on dietary restriction alone, while 14 (12 %) and 21 (17.9 %) were taking metformin or glibenclamide, respectively. 62 patients (53 %) were under treatment with combination of metformin and glibenclamide, and 13 subjects (11.1 %) were on combination therapy with metformin and insulin. Mean ± Standard deviation (SD) of diabetes duration was 8.9 ± 6.6 years. The number of smokers, hypertensive and hyperlipidemic subjects in diabetes group were 10 (8.5 %), 60 (51.3 %) and 108 numbers (92.3 %), respectively.

The baseline characteristics of diabetic patients according to glycemic status are shown in Table 2. The poor control group were dominantly women, had higher levels of FBS, TC, TG, LDL-C, HbA1c, CIMT, WC, BMI and longer duration of diabetes with lower TAS levels and ABI compared to the good control group (Table 2).

**Table 2:** Baseline characteristics of 117 diabetic patients according to glycemic status

Variable	Good control diabetics *N* = 53	Poor control diabetics *N* = 64	*P* value
Age (year)	54.3 ± 8	53 ± 8.5	0.50
Female (n (%))	26 (42.6)	35 (57.4)	0.58
Diabetes duration (year)	6.9 ± 5	10 ± 6.5	0.004*
SBP (mmHg)	133 ± 19	135 ± 17	0.44
DBP (mmHg)	77 ± 11	79 ± 9	0.20
BMI (kg/m^2^)	27.7 ± 4	28 ± 4	0.58
WC (cm)	94.8 ± 9	94.7 ± 12	0.92
TC (mg/dl)	166 ± 32	184 ± 47	0.02*
TG (mg/dl)^a^	174 ± 97	211 ± 115	0.02*
LDL-C (mg/dl)	89 ± 21	101 ± 28	0.01*
HDL-C (mg/dl)	42 ± 9	41 ± 10	0.72
FBS (mg/dl)	126 ± 27	199 ± 65	<0.001*
HbA1c (%)	6.5 ± 0.7	9.3 ± 1.3	<0.001*
TAS (mmol/l)^a^	3.23 ± 0.68	3.04 ± 0.52	0.30
Max. CIMT (mm)^a^	1.25 ± 0.83	1.41 ± 1.08	0.30
ABI	1.16 ± 0.11	1.11 ± 0.11	0.03*

*SBP* systolic blood pressure, *DBP* diastolic blood pressure, *BMI* body mass index, *WC* waist circumference, *TC* total cholesterol, *TG* triglycerides, *LDL-C* low density lipoprotein cholesterol, *HDL-C* high density lipoprotein cholesterol, *FBS* fasting blood sugar, *HbA1c* glycated hemoglobin A1c, *TAS* total antioxidant status, *CIMT* carotid intima media thickness, *ABI* ankle brachial index

*P ≤ 0.05 was considered as statistically significant

^a^For variables with abnormal distribution was used nonparametric analytic tests

In diabetes group, TAS was similar in males and females (mean 3.14 mmol/l in men vs. 3.11 mmol/l in women. Inter-quartile range of TAS that determines difference between quartiles 75 and 25 was also similar in men [3.23–2.86 mmol/l] and women [3.16–2.80 mmol/l]; (*p* = 0.79). In addition TAS levels decreased with age in men (3.17 vs. 3.13) and increased in females (3.10 vs 3.12) but without significant differences;(*p* = 0.82, and *p* = 0.93, respectively).

The correlation between indices of glycemic status and TAS was a weak negative correlation (*r* = −0.10) with nonsignificant statistical difference (*p* = 0.30). This correlation for CIMT, and ABI was *r* = 0.09, and *r* = −0.16, respectively. The difference in all of these correlations was not statistically significant, *p* = 0.30, and *p* = 0.09, respectively. Coefficients of univariate and multivariate regression models between TAS, CIMT, and ABI as outcomes with demographic characteristics, BMI, WC, hypertension, and hyperlipidemia as covariates according to glycemic status (≤7.5/>7.5) are shown in Table 3.

**Table 3:** Associatieon between diabetic control (HbA1c) with surrogated markers of subclinical atherosclerosis and total antioxidant score in Univariate and multivariable logistic regression models

Independent variables	CIMT (≤0.8 / >0.8)		ABI (≤0.9 / >0.9)		TAS (≤ Median / > Median)	
	Univariate odds ratio CI 95 %	Multivariate odds ratio CI 95 %	Univariate odds ratio CI 95 %	Multivariate odds ratio CI 95 %	Univariate odds ratio CI 95 %	Multivariate odds ratio CI 95 %
HbA1c %	1.12 (0.90 – 1.40)	1.17 (0.87 – 1.56)	1.18 (0.88 – 1.58)	1.16 (0.87 – 1.55)	0.94 (0.72 – 1.22)	0.89 (0.67 – 1.17)
Age (year)	1.09 (1.03 – 1.15)	1.10 (1.03 – 1.18)	0.98 (0.92 – 1.05)	-	1.01 (0.96 – 1.07)	-
Sex (F/M)	0.83 (0.37 – 1.84)	-	0.42 (0.13 – 1.31)	0.51 (0.15 – 1.72)	0.68 (0.28 – 1.66)	-
Diabetes duration (year)	1.06 (0.99 – 1.13)	0.98 (0.91 – 1.07)	0.99 (0.90 – 1.08)	-	0.98 (0.92 – 1.06)	-
BMI (kg/m^2^)	0.92 (0.83 – 1.02)	1.00 (0.83 – 1.20)	0.89 (0.77 – 1.03)	0.92 (0.80 – 1.07)	1.01 (0.90 – 1.12)	-
WC (cm)	0.94 (0.90 – 0.99)	0.91 (0.83 – 0.99)	0.98 (0.94 – 1.03)	-	1.03 (0.98 – 1.08)	-
Hypertension (Y/N)	2.87 (1.24 – 6.60)	4.20 (1.41 – 12.51)	1.53 (0.51 – 4.62)	-	1.40 (0.57 – 3.42)	-
High LDL-C (Y/N)	3.94 (1.19 – 13.01)	3.77 (0.84 – 16.86)	0.47 (0.06 – 3.86)	-	2.86 (0.79 – 10.30)	3.26 (0.86 – 12.33)

*CIMT* carotid intima media tickness, *ABI* ankle brachial index, *TAS* total antioxidant status, *BMI* body mass index, *WC* waist circumference, *Hypertension* high blood pressure ≥140/90 mmHg or histoey of hypertension, *High LDL-C* LDL-C ≥ 130 mg/dl

The variables with P< 0.2 in univarIable models enter in multivariable models

## Discussion

We found significantly lower TAS leves and ABI values in diabetic patients. In addition CIMT was significantly higher in diabetics compared to the control group. After dividing diabetics into two subgroups according to the glycemic status, we did not find any significant influence of glycemic status on TAS and ABI. However, glycemic status showed a significant influence on the association between age and diabetes duration with CIMT. By increasing age and duration of diabetes, accretion of CIMT was significantly more in poor glycemic group versus well controlled diabetics.

The excess risk for CVD mortality in diabetics can be justified to some extent by increased prevalence of traditional risk factors such as hypertension, obesity and hyperlipidemia in this group [21]. For example, the results of the United Kingdom Prospective Diabetes Study (UKPDS) showed a significant association between increased risk of CVD in diabetics with hyperlipidemia and increased HbA1c levels [22]. However, an important contributory factor for excess risk of CVD in diabetes would be oxidative stress which is associated with increased lipid peroxidation [2]. Hyperglycemia associated with hyperlipidemia is a causative factor for increased lipid peroxidation in diabetics [23]. This is supported by impaired antioxidant status and increased oxidative damage to lipids in type 2 diabetes. In this regard use of antioxidant nutrients is suggested as adjuvants in the prevention of CVD in T2DM [24]. All of the different parameters of hyperglycemia, acute or chronic, contribute to the vascular damage, likely via generation of reactive oxygen species. We observed significantly higher levels of dyslipidemia parameters in our poor glycemic group than those with good glycemic control. This is a substantial finding as the results of UKPDS have proven that good control of diabetes corrects lipid abnormalities in almost 65 % of these subjects [25]. So, strict glycemic control should be the first objective to prevent CVD associated with oxidative damage to lipids in T2DM.

Of various markers for oxidative stress detection in diabetes, total Antioxidant Status (TAS) considers the cumulative action of all the antioxidants present in plasma and body fluids; and provides an integrated measure of oxidative stress rather than simply assessing sum antioxidants [26]. It is proven that measurement of TAS is of paramount importance in diagnosis of oxidative state [26]. In addition TAS is a useful marker to determine prognosis and guide antioxidant therapy [26].

The level of TAS in the poor glycemic group in our study, was lower than the well controlled glycemic ones; although with a non- significant statistical difference, which was in agreement with previous reports [27]. In a study by Willems et al. in type 1 diabetic patients, there was no difference in TAS levels between the 2 subgroups with and without subclinical complications; however both of these subgroups were glycemically well controlled [28].

We suggest that the association of low TAS levels with poor glycemic status would justify prescription of antioxidant supplies as dietary supplements in the treatment of poor glycemic diabetics. Moreover, hypertension and hyperlipidemia which very often coexist with type 2 diabetes produce oxidative stress and contribute to low TAS levels in these patients [29, 30]. While antioxidant therapy is advantageous in secondary prevention of oxidative damage, it laso assists in control of hypertension and dyslipidemia [31]. Antioxidant therapy also hinders progression of vascular damage in diabetics by helping optimal control of glucose in these patients [31].

An interesting finding of this study was an age dependent increase in TAS among women who were metabolically controlled; although this favourable effect was not observed in men.

Sundaram et al. have demonstrated an age-dependent reduction in total antioxidant capacity, and an age independent increase in lipid profile and oxidative damage in elderly diabetics [32]. In the elderly diabetics, age-related perturbations of TAS would contribute significantly to the development of cardiovascular complications. This idea is worth to be testified in complementary larger scale trials.

The association between increased CIMT and CVD is well established [33]. CIMT is increased in patients with T2DM [34]. In the Epidemiology of Diabetes Interventions and Complications (EDIC) study, CIMT was measured 18 months after the Diabetes Control and Complications Trial (DCCT) and the investigators did not find a significant correlation between mean levels of HbA1c with CIMT during the study period. However when this measurement was extended 6 years later, a significant positive association was observed in both sexes between mean HbA1c levels and CIMT [35]. After 18 years, this association was still consistent and also more robust in diabetic women. This may be related to stronger atherosclerotic effect of CVD risk factors in women compared to men.

There are various reports in the literature regarding the association of CIMT with risk factors of carotid atherosclerosis. Some authors [36, 37] were unable to find a statistically significant correlation between CIMT and CVD risk factors in diabetic patients. Other investigators nonetheless, have reported a significant association between CIMT and age, sex, smoking, blood pressure, body mass index [38–40] and the presence of diabetes or glucose intolerance [34, 41, 42]. In our study, like the findings of two other research groups [36, 37], we did not observe a significant correlation between CIMT and gender, hyperlipidemia, hypertension, obesity, or HbA1c. However, we found just a significant association between age and duration of diabetes in poor glycemic group. It is probably because diabetes itself is of crucial importance for the development of atherosclerosis because of clustering of multiple interrelated metabolic disturbances over- shadowing the contribution of other risk factors.

Peripheral Artery Disease (PAD), a manifestation of atherosclerosis in the lower limbs occurs with more than four-folds increased risk in diabetics. With each 1 % increase in HbA1c, the risk of PAD increases 28 %. Currently the most widely accepted test to assess PAD is the ABI. Decreased ABI is a major risk factor of CVD mortality [43] and low ABI (≤0.9) is an indicator of PAD [15]. These observations in diabetics may simply reflect the severity of diabetes due to its long duration and poor glycemic control. In general, risk factors for PAD are similar to those of coronary artery disease [44, 45].

There are diverse findings regarding the effect of glycemic control on PAD in T2DM. Some studies show a significant associatin between fasting serum glucose and HbA1c with PAD; in contrast to others [46, 47].

Although, there is no conclusive evidence to suggest that optimal glycemic control lowers the risk of PAD, in the presence of an increased risk of cardiovascular events, it is logical that optimal glycemic control would be desirable in patients with PAD. In our patients, there was no significant difference in ABI between two groups after adjusted analysis for glycemic control. One reason of this finding may be the absence of ABI ≤ 0.9 in our patients (The average of ABI within both good and poor glycemic groups was 1.0).

One limitation of the study was that we did not assess postprandial hyperglycemia and individual antioxidant intake of the participants. In addition, due to the cross sectional design of the study and small sample size, we were not able to evaluate the influence of age, sex, diabetes duration, obesity, hypertension and hyperlipidemia on oxidative stress and surrogate measures of subclinical atherosclerosis comprehensively in these patients. Our results may be confounded as well by anti-diabetic, anti-hypertensive and lipid lowering medications; almost all of them have potent antioxidant effects and may have an independent effect on TAS and surrogate SA markers [48–50]. Complementary studies are needed in order to confirm our findings.

## Conclusions

Our observations are in favour of the hypothesis that persistent hyperglycemia leads to decreased TAS levels and worse CIMT which is more pronounced in diabetics with poor glycemic control.

Considering the conflicting outcomes of different studies it is suggested that further cohort studies with larger number of patients are required to find out the interrelationship and contribution of various risk factors of atherosclerosis in diabetic patients. As expected the blood pressure, lipid profile, weight, oxidative stress and vascular stiffness in our patients with poor glycemic control were worse than well controlled ones. Increased CIMT, impaired ABI, and decreased TAS levels are considered to be the initial steps in cardiovascular complications of diabetes, which may be reversible. Thus in diabetic patients we suggest assessment of TAS levels concomitant with CIMT measurement for early initiation of antioxidant therapy. In addition, it is necessary to initiate primary and secondary preventive measures such as including plenary life style modification and use of lipid-lowering, antihypertensive and glucose-lowering drugs to mitigate the devastating consequences of diabetes leading to CVD.

## Footnotes

1 National Cholesterol Education Program-Adult Treatment Panel III 

## References

[1] Ceriello A, Motz E (2004). Is oxidative stress the pathogenic mechanism underlying insulin resistance, diabetes, and cardiovascular disease? The common soil hypothesis revisited. Am Heart Assoc.

[2] Rahimi R, Nikfar S, Larijani B, Abdollahi M (2005). A review on the role of antioxidants in the management of diabetes and its complications. Biomed Pharmacother.

[3] Saeidnia S, Abdollahi M (2013). Toxicological and pharmacological concerns on oxidative stress and related diseases. Toxicol Appl Pharmacol.

[4] Barr EL, Zimmet PZ, Welborn TA, Jolley D, Magliano DJ, Dunstan DW (2007). Risk of cardiovascular and all-cause mortality in individuals with diabetes mellitus, impaired fasting glucose, and impaired glucose tolerance: the Australian Diabetes, Obesity, and Lifestyle Study (AusDiab). Circulation.

[5] Murray CJ, Lopez AD (1997). Alternative projections of mortality and disability by cause 1990–2020: Global Burden of Disease study. Lancet.

[6] UK Prospective Diabetes Study (UKPDS) Group (1998). Intensive blood glucose control with sulphonylureas or insulin compared with conventional treatment and risk of complications in patients with type 2 diabetes (UKPDS 33). Lancet.

[7] Tabatabaei-Malazy O, Qorbani M, Samavat T, Sharifi F, Larijani B, Fakhrzadeh H (2014). Prevalence of dyslipidemia in Iran: a systematic review and meta-analysis study. Int J Prev Med.

[8] Herman WH, Fajans SS (2010). Hemoglobin A1c for the diagnosis of diabetes: practical considerations. Pol Arch Med Wewn.

[9] American Diabetes Association (2014). Diagnosis and classification of diabetes mellitus. Diabetes Care.

[10] Bakhtiari S, Bigom Taheri J, Bakhshi M, Mortazavi H, Shah Hoseini A, Vahid Dastjerdi E (2012). Effect of vitamin C on salivary total antioxidant capacity in smokers. Iran J Pharm Res.

[11] Wang Y, Chun OK, Song WO (2013). Plasma and dietary antioxidant status as cardiovascular disease risk factors: a review of human studies. Nutrients.

[12] Wang Y, Yang M, Lee SG, Davis CG, Kenny A, Koo SI (2012). Plasma total antioxidant capacity is associated with dietary intake and plasma level of antioxidants in postmenopausal women. J Nutr Biochem.

[13] Naghavi M, Falk E, Hecht HS, Jamieson MJ, Kaul S, Berman D (2006). From vulnerable plaque to vulnerable patient--Part III: Executive summary of the Screening for Heart Attack Prevention and Education (SHAPE) Task Force report. Am J Cardiol.

[14] Lorenz MW, Markus HS, Bots ML, Rosvall M, Sitzer M (2007). Prediction of clinical cardiovascular events with carotid intima-media thickness: a systematic review and meta-analysis. Circulation.

[15] Dobby AV, Anand SS (2005). Sensitivity and specificity of the ankle-brachial index to predict future cardiovascular outcomes: a systematic review. Arterioscler Thromb Vasc Biol.

[16] Holman RR, Paul SK, Bethel MA, Matthews DR, Neil HAW (2008). 10-year follow-up of intensive glucose control in type 2 diabetes. N Engl J Med.

[17] Duckworth W, Abraira C, Moritz T, Reda D, Emanuele N, Reaven PD (2009). Glucose control and vascular complications in veterans with type 2 diabetes. N Engl J Med.

[18] Friedewalds WT, Levy RI, Fredrickson DS (1972). Estimation of the concentration of low density lipoprotein cholesterol in plasma without the use of preparative ultracentrifuge. Clin Chem.

[19] Talwalkar PG, Sreenivas CG, Gulati A, Baxi H (2013). Journey in guidelines for lipid management: From adult treatment panel (ATP)-I to ATP-III and what to expect in ATP-IV. Indian J Endocrinol Metab.

[20] Bogousslavsky J, Melle GV, Regli F (1988). The Lausanne stroke registry. Analysis of 1000 consecutive patients with first stroke. Stroke.

[21] O’Donnell CJ, Elosua R (2008). Cardiovascular risk factors. Insights from Framingham Heart Study. Rev Esp Cardiol.

[22] Turner RC (1998). The UK, prospective diabetic study: a review. Diabetic Care.

[23] Manohar SM, Vaikasuvu SR, Deepthi K, Sachan A, Narasimha SR (2013). An association of hyperglycemia with plasma malondialdehyde and atherogenic lipid risk factors in newly diagnosed type 2 diabetic patients. J Res Med Sci.

[24] Song Y, Cook NR, Albert CM, Van Denburgh M, Manson JE (2009). Effects of vitamins C and E and beta-carotene on the risk of type 2 diabetes in women at high risk of cardiovascular disease: a randomized controlled trial. Am J Clin Nutr.

[25] Ahuja A, Roopakala MS, Silvia WD, Sanjay Reddy S, Kumar KMP (2011). Glycated hemoglobin, dyslipidemia and risk of atherosclerosis in type 1 diabetic patients. Int J Diabetes Dev Ctries.

[26] Tabatabaei-Malazy O, Nikfar S, Larijani B, Abdollahi M (2014). Influence of ascorbic acid supplementation on type 2 diabetes mellitus in observational and randomized controlled trials; a systematic review with meta-analysis. J Pharm Pharm Sci.

[27] Cakatay U (2005). Protein oxidation parameters in type 2 diabetic patients with good and poor glycaemic control. Diabetes Metab.

[28] Al-Nimer MS, Al-Ani FS, Ali FS (2012). Role of nitrosative and oxidative stress in neuropathy in patients with type 2 diabetes mellitus. J Neurosci Rural Pract.

[29] Moriel P, Plavnik FL, Zanella MT, Bertolami MC, Abdalla DS (2000). Lipid peroxidation and antioxidants in hyperlipidemia and hypertension. Biol Res.

[30] García-Fontana B, Morales-Santana S, Longobardo V, Reyes-García R, Rozas-Moreno P, García-Salcedo JA (2015). Relationship between proinflammatory and antioxidant proteins with the severity of cardiovascular disease in type 2 diabetes mellitus. Int J Mol Sci.

[31] Steiner G (2000). Lipid intervention trials in diabetes. Diabetes Care.

[32] Prasad K, Dhar I (2014). Oxidative stress as a mechanism of added sugar-induced cardiovascular disease. Int J Angiol.

[33] Weber LA, Cheezum MK, Reese JM, Lane AB, Haley RD, Lutz MW (2015). Cardiovascular imaging for the primary prevention of atherosclerotic cardiovascular disease events. Curr Cardiovasc Imaging Rep.

[34] Hunt KJ, Williams K, Rivera D, O'Leary DH, Haffner SM, Stern MP (2003). Elevated carotid artery intima media thickness levels in individuals who subsequently develop type 2 diabetes. Arterioscler Thromb Vasc Biol.

[35] Nathan DM, Lachin J, Cleary P, Orchard T, Brillon DJ, Backlund JY (2003). Intensive diabetes therapy and carotid intimamedia thickness in type 1 diabetes mellitus. N Engl J Med.

[36] Herinirina NF, Rajaonarison LH, Herijoelison AR, Ahmad A (2015). Thickness of carotid intima-media and cardiovascular risk factors. Pan Afr Med J.

[37] Dabas A, Yadav S, Gupta VK (2014). Lipid profile and correlation to cardiac risk factors and cardiovascular function in type 1 adolescent diabetics from a developing country. Int J Pediatr.

[38] Rashid SA, Mahmud SA (2015). Correlation between carotid artery intima-media thickness and luminal diameter with body mass index and other cardiovascular risk factors in adults. Sultan Qaboos Univ Med J.

[39] Nagasawa SY, Ohkubo T, Masaki K, Barinas-Mitchell E, Miura K, Seto T (2015). Associations between inflammatory markers and subclinical atherosclerosis in middle-aged white, Japanese-American and Japanese men: the ERA-JUMP Study. J Atheroscler Thromb.

[40] Tabatabaei-Malazy O, Fakhrzadeh H, Sharifi F, Mirarefin M, Badamchizadeh Z, Larijani B (2012). Gender differences in association between metabolic syndrome and carotid intima media thickness. J Diabetes Metab Disord.

[41] Fakhrzadeh H, Alatab S, Sharifi F, Mirarefein M, Badamchizadeh Z, Ghaderpanahi M (2012). Carotid intima media thickness, brachial flow mediated dilation and previous history of gestational diabetes mellitus. J Obstet Gynaecol Res.

[42] Koskinen J, Kahonen M, Viikari JSA, Taittonen L, Laitinen T, Ronnemaa T (2009). Conventional cardiovascular risk factors and metabolic syndrome in predicting carotid intima-media thickness progression in young adults: the cardiovascular risk in young Finns study. Circulation.

[43] Benjamin I, Brown N, Burke G, Correa A, Houser SR, Jones DW (2015). American Heart Association Cardiovascular Genome-Phenome Study: foundational basis and program. Circulation.

[44] Bianchi C, Penno G, Pancani F, Civitelli A, Piaggesi A, Caricato F (2007). Non-traditional cardiovascular risk factors contribute to peripheral arterial disease in patients with type 2 diabetes. Diabetes Res Clin Pract.

[45] Jude EB, Eleftheriadou I, Tentolouris N (2010). Peripheral arterial disease in diabetes-a review. Diabet Med.

[46] Thiruvoipati T, Kielhorn CE, Armstrong EJ (2015). Peripheral artery disease in patients with diabetes: epidemiology, mechanisms, and outcomes. World J Diabetes.

[47] Muntner P, Wildman RP, Reynolds K, Desalvo KB, Chen J, Fonseca V (2005). Relationship between HbA1c level and peripheral arterial disease. Diabetes Care.

[48] Behzad M, Negah R, Sureer B, Neda R (2007). A review of thiazolidine diones and metformin in the treatment of type 2 diabetes with focus on cardiovascular complications. Vasc Health Risk Manag.

[49] Li W, Chen Y, Li S, Guo X, Zhou W, Zeng Q (2014). Agonistic antibody to angiotensin II type 1 receptor accelerates atherosclerosis in ApoE−/− mice. Am J Transl Res.

[50] Lim S, Barter P (2014). Antioxidant effects of statins in the management of cardiometabolic disorders. J Atheroscler Thromb.

